# Cellular mechanisms of the 5-HT_7_ receptor-mediated signaling

**DOI:** 10.3389/fnbeh.2014.00306

**Published:** 2014-10-01

**Authors:** Daria Guseva, Alexander Wirth, Evgeni Ponimaskin

**Affiliations:** Department of Cellular Neurophysiology, Hannover Medical SchoolHannover, Germany

**Keywords:** serotonergic signaling, G-protein coupled receptors, serotonin 5-HT_7_ receptor, heterotrimeric G-protein, oligomerization, palmitoylation

## Abstract

Serotonin (5-hydroxytryptamine or 5-HT) is an important neurotransmitter regulating a wide range of physiological and pathological functions via activation of heterogeneously expressed 5-HT receptors. The 5-HT_7_ receptor is one of the most recently described members of the 5-HT receptor family. Functionally, 5-HT_7_ receptor is associated with a number of physiological and pathological responses, including serotonin-induced phase shifting of the circadian rhythm, control of memory as well as locomotor and exploratory activity. A large body of evidence indicates involvement of the 5-HT_7_ receptor in anxiety and depression, and recent studies suggest that 5-HT_7_ receptor can be highly relevant for the treatment of major depressive disorders. The 5-HT_7_ receptor is coupled to the stimulatory G_s_-protein, and receptor stimulation results in activation of adenylyl cyclase (AC) leading to a rise of cAMP concentration. In addition, this receptor is coupled to the G_12_-protein to activate small GTPases of the Rho family. This review focuses on molecular mechanisms responsible for the 5-HT_7_ receptor-mediated signaling. We provide detailed overview of signaling cascades controlled and regulated by the 5-HT_7_ receptor and discuss the functional impact of 5-HT_7_ receptor for the regulation of different cellular and subcellular processes.

## General principles of G-protein coupled receptor signaling

G-protein coupled receptors (GPCRs) represent the largest and most diverse superfamily of transmembrane receptors divided into five different families: rhodopsin, secretin, glutamate, adhesion and frizzled receptors (Bjarnadóttir et al., 2006). Initial studies with first discovered GPCRs, bovine rhodopsin and β2 adrenergic receptor, arouse great interest in the field of GPCRs, whose structures and functions became a subject of extensive research (Nathans and Hogness, 1983; Dixon et al., 1986). All these receptors function as signal-transducers by translating extracellular stimuli into intracellular responses resulting in multiple physiological as well as pathophysiological responses (Thompson et al., 2008). All known GPCRs consist of an extracellular amino-terminus, seven membrane-spanning α-helices (for which reason they are often referred to as 7 transmembrane receptors), and an intracellular carboxyl-terminus. Hence GPCR activity is induced by many different ligands, the mechanism of sensing ligands and transducing signals are highly variable (reviewed in Kristiansen, 2004). According to the “allosteric ternary complex model”, GPCRs exist in equilibrium between an inactive and active state (Christopoulos and Kenakin, 2002), explaining the agonist-independent, constitutive activity of some receptors (Seifert and Wenzel-Seifert, 2002).

## Heterotrimeric G-proteins

Heterotrimeric G-proteins are the main downstream effectors of GPCRs acting as molecular switches by turning on intracellular downstream signaling cascades. They consist of three subunits, α, β and γ and are divided into four subgroups according to the structural and functional similarities of the Gα subunit. The members of the stimulatory Gα_s_ family stimulate adenylyl cyclases (ACs), whereas inhibitory Gα_i_ proteins inhibit ACs. The Gα_q_ class of G-proteins couples to phospholipase Cβ (PLCβ), while Gα_12_ family members activate Rho guanine-nucleotide exchange factors (Rho GEFs; Kristiansen, 2004). To date at least 16 different genes encoding Gα subunits, 5 genes encoding Gβ subunits and 12 different genes encoding Gγ subunits have been discovered. Although not all subunits do interact with each other, the diversity of heterotrimeric G-proteins is still enormous, and this represents an additional level of complexity by the regulation of multiple signaling pathways (Cabrera-Vera et al., 2003).

Heterotrimeric G-proteins become activated by GPCRs *via* complex conformational changes, which are also facilitated by Gβγ dimers (Ford et al., 1998). Upon discovery of the heterotrimeric G-proteins, they were thought to conduct signals exclusively *via* Gα-subunits. Later on, Gβγ dimer has also been shown to directly modulate downstream effectors. First identified downstream target of Gβγ dimer was G-protein coupled inward rectifier potassium (GIRK) channel (Logothetis et al., 1987). Nowadays, a list of downstream effectors regulated by Gβγ dimers is permanently extending (Woehler and Ponimaskin, 2009).

In parallel with this classical G-protein mediated GPCR signaling, non-classical (G-protein independent) signaling became obvious during the last decade. This type of signaling will be also discussed below.

## G-protein independent signaling

Beside the canonical GPCR signaling pathways *via* heterotrimeric G-proteins, GPCRs can participate in non-canonical, G-protein independent signaling. Main players of the G-protein independent signaling are arrestins - a small family of cytosolic adaptor proteins consisting of four members (Krupnick and Benovic, 1998). In contrast to arrestin 1 and arrestin 4 (X arrestin), which are primary involved in adaption processes of opsins in rods or cones, arrestin 2 and 3 (β-arrestin 1 and 2) are ubiquitously expressed and can interact with different GPCRs (Lefkowitz and Shenoy, 2005). Shortly after receptor stimulation, the C-terminal tail of a GPCR often becomes substrate for the phosphorylation by G-protein coupled receptor kinases (GRKs; Gehret and Hinkle, 2010). Phosphorylated receptors display a high affinity for β-arrestin 1 and 2, which hinder interactions between receptor and heterotrimeric G-protein resulting in desensitization and damping of G-protein dependent signaling (Perry et al., 2002). However, differently than thought at the beginning, arrestins not only switch-off the GPCR-signaling, but can also lead to the activation of alternative signaling pathways. Thus, β-arrestins serve as a signaling hub, linking activated GPCRs to multiple (G-protein independent) signaling pathways such as receptor trafficking as well as in extending GPCR mediated signaling to non-receptor tyrosine kinases (nRTKs) like proto-oncogene c-Src (c-Src) and mitogen-activated protein kinases (MAPK) signaling pathways.

## 5-HT_7_ receptor: physiological functions and distribution in the brain

The 5-HT_7_ receptor is one of the most recently discovered members of the serotonin receptor family, which was cloned in 1993 independently by researchers in three laboratories (Bard et al., 1993; Lovenberg et al., 1993; Ruat et al., 1993). The 5-HT_7_ receptor gene is located on human chromosome 10q23.3-q24.3 with an open reading frame containing 1335 base pairs and encoding a protein of 445 amino acids (Bard et al., 1993). The 5-HT_7_ receptor is broadly expressed in the central nervous system including spinal cord (Dogrul and Seyrek, 2006), thalamus, hypothalamus, hippocampus, prefrontal cortex, and the amygdala where it is expressed in both neurons and glial cells (Hedlund and Sutcliffe, 2004; Thomas and Hagan, 2004; Russo et al., 2005). Significant density of 5-HT_7_ receptor was observed in raphe nuclei area. In contrast, receptor expression level detected in putamen and cerebellum was relatively low (Horisawa et al., 2013). The 5-HT_7_ receptor is also expressed in the suprachiasmatic nucleus, and one of the first functions proposed for the 5-HT_7_ receptor was the regulation of sleep/wake cycles (Lovenberg et al., 1993). Functional analysis demonstrated association of the 5-HT_7_ receptor with central processes such as learning and memory, including specific aspects of hippocampus-dependent information processing (Hedlund and Sutcliffe, 2004; Ballaz et al., 2007; Eriksson et al., 2008; Gasbarri et al., 2008; Hedlund, 2009). Moreover, 5-HT_7_ receptor can be implicated in several neurological diseases (Hedlund and Sutcliffe, 2004; Thomas and Hagan, 2004). It has been shown that pharmacological blockade or knock-down of the 5-HT_7_ receptor induces antidepressant-like behavior in animal models (Guscott et al., 2005; Hedlund et al., 2005; Wesołowska et al., 2007). In addition, certain antidepressants may act directly on the 5-HT_7_ receptor (Mullins et al., 1999), suggesting this receptor as a novel target by the treatment of depression (Hedlund, 2009; Mnie-Filali et al., 2009). Analysis of mRNA expression level revealed that the amount of 5-HT_7_ gene transcripts in the dorsolateral prefrontal cortex of schizophrenic patients was increased, demonstrating that 5-HT_7_ receptor can also be associated with schizophrenia (East et al., 2002; Pouzet et al., 2002; Ikeda et al., 2006).

So far, three splice variants of the 5-HT_7_ receptor have been identified in human, including 5-HT_7(a),_ 5-HT_7(b),_ 5-HT_7(d)_, three in mouse - 5-HT_7(a),_ 5-HT_7(b),_ 5-HT_7(d)_, and four in rat - 5-HT_7(a),_ 5-HT_7(b),_ 5-HT_7(c)_, 5-HT_7(e)_ (Heidmann et al., 1997; Liu et al., 2001). These splice variants differ only in their short carboxyl-terminal amino acid sequence. Receptor isoforms have altered patterns of tissue distribution, while no difference in their pharmacological properties and coupling to ACs was observed (Heidmann et al., 1997, 1998; Krobert et al., 2001). The human 5-HT_7(d)_ receptor represents an exception, because this isoform possesses a differential pattern of receptor internalization which can affect receptor-mediated signaling (Guthrie et al., 2005). In this regard, 5-HT_7(d)_ receptor was constitutively internalized in the absence of agonist suggesting that its carboxyl-terminal tail, which is the longest among known human 5-HT_7_ receptor isoforms, may contain a motif that interacts with cellular transport machinery that is distinct from 5-HT_7(a)_ and 5-HT_7(b)_ receptors.

## Gα_s_ signaling mediated by the 5-HT_7_ receptor

**Figure 1:**
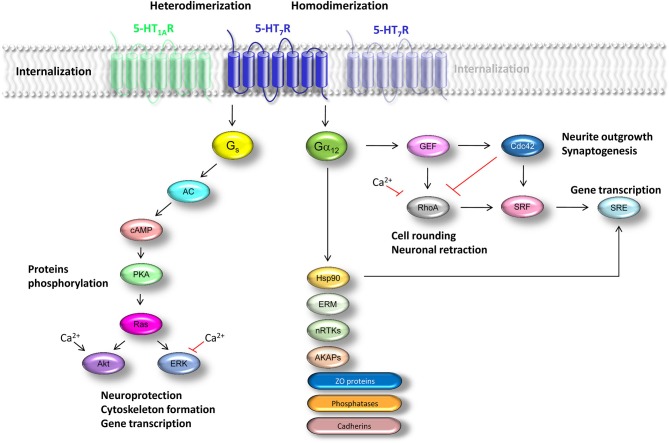
**Schematic representation of signaling pathways regulated by the 5-HT_7_ receptor**. Effects mediated by G_s_-proteins are in the left section. Summary of the G_12_-mediated signaling processes is shown in the right section. Abbreviations: GIRK—G-protein coupled inward rectifier potassium channel; AC—adenylyl cyclase; cAMP—cyclic adenosine monophosphate; PKA—protein kinase A; ERK—extracellular signal-regulated kinases; Akt—protein kinase B, Hsp90—heat shot shock protein 90; ERM—proteins of the ezrin-radixin-moesin family; GEF—guanine-nucleotide exchange factor (represented by the leukemia-associated RhoGEF LARG and p115Rho); nRTKs—non-receptor tyrosine kinases; AKAPs—A-kinase anchoring proteins; ZO—zona occludens proteins; SRF—serum response factor; SRE—serum response element.

The canonical signaling pathway of the 5-HT_7_ receptor is activation of G_s_-protein which in turn can activate different AC isoforms (Shen et al., 1993). ACs show a unique tissue distribution as well as regulatory properties (Krupinski et al., 1989; Bakalyar and Reed, 1990; Premont et al., 1996). *In vitro*, all known AC isoforms are sensitive to the G_s_ activation (Cooper et al., 1995; Taussig and Gilman, 1995; Sunahara et al., 1996). In contrast, it has been demonstrated that Ca^2+^/calmodulin-stimulated neural-specific isoforms AC1 and AC8 are insensitive to G_s_ *in vivo* (Impey et al., 1994; Wayman et al., 1994; Nielsen et al., 1996), and that 5-HT_7(a)_ receptor isoform can stimulate AC1 and AC8 by increasing intracellular Ca^2+^ concentration (Baker et al., 1998). The coupling between 5-HT_7_ receptor and G_s_-protein results in increased AC activity leading to production of cAMP, which in turn activates protein kinase A (PKA) thereby inducing phosphorylation of different target proteins (Figure 1). This results in activation of multiple downstream signaling cascades, including Ras-dependent and Rap1-independent activation of the neuroprotective extracellular signal-regulated kinases (ERK) and Akt (protein kinase B) pathways (Errico et al., 2001; Johnson-Farley et al., 2005). Noteworthy, 5-HT_7_ receptor-mediated activation of Akt requires increases both in [cAMP] and intracellular [Ca^2+^], while activation of ERK is inhibited by Ca^2+^ (Figure 1). However, neither an influx of extracellular Ca^2+^ nor release of intracellular Ca^2+^ stores was required for 5-HT_7_ receptor-mediated activation of ERK in cultured primary hippocampal neurons (Lin et al., 2003). The authors of this study also demonstrated that increase in cAMP concentration causes activation of ERK in neurons *via* a pathway independent of PKA and Raf-1 (Li et al., 1991; Kyriakis et al., 1992). It is widely accepted, that intracellular pathways regulating ERK1/2 and Akt signaling are involved in actin filament reorganization. On the other hand, studies with LM2 cells, which are able to invade into the lung tissue *in vivo*, revealed no significant inhibition in cell motility after Ras-ERK pathway blockade, while PI3K pathways was critically involved in regulation of motility of LM2 cells (Choi and Helfman, 2014). It has been also shown that activation of PI3K activity alone is sufficient to remodel actin filaments and to increase cell migration through the activation of Akt in chicken embryo fibroblast (Qian et al., 2004). Thus, 5-HT_7_ receptor-mediated activation of G_s_-protein can be involved in the activation of effector molecules regulating the cellular motility and cytoskeleton formation.

## Gα_12_ signaling mediated by the 5-HT_7_ receptor

In our previous studies we have demonstrated that 5-HT_7_ receptor is coupled not only to the G_s_-protein, but can also activate G_12_-protein (Figure 1; Kvachnina et al., 2005; Kobe et al., 2012).

The G_12_-proteins have been shown to activate multiple signaling pathways, and their prominent downstream effectors are members of the Rho family of small GTPases (Rho, Rac, and Cdc42). The G_12_-protein can modulate the activity of Rho GTPases by activation of guanine-nucleotide exchange factor (GEF) p115Rho which was the first identified downstream effector of Gα_12_ proteins (Hart et al., 1998; Kozasa et al., 1998). Later on, plethora of additional downstream targets of G_12_-proteins has been discovered. In addition to other RhoGEFs, such as leukemia-associated RhoGEF (LARG) and RhoGEF homologs in *Caenorhabditis elegans*, regulator of G-protein signaling (RGS) family members, proteins of the ezrin-radixin-moesin (ERM) family, nRTKs, protein phosphatases, A-kinase anchoring proteins (AKAPs), zona occludens proteins and heat shot shock protein 90 (Hsp90) have been identified to directly interact with heterotrimeric G_12_-protein (Figure 1; Hiley et al., 2006; Kelly et al., 2007). The Gα_12_ subunit can also interact with C-terminal parts of cadherins leading to release of β-catenin into cytoplasm and nucleus, thus triggering gene transcription (Meigs et al., 2001).

In case of 5-HT_7_ receptor, it has been reported that receptor-mediated stimulation of G_12_-protein results in Rho-dependent activation of a transcription factor, serum response factor (SRF), which binds to the serum response element (SRE; Figure 1). Noteworthy, stimulation of 5-HT_7_ receptor led to the dose-dependent increase in SRE-driven gene expression even in the presence of a PKA-inhibitor or pertussis toxin (PTX), suggesting a receptor-mediated SRE activation in a PKA-independent manner (Kvachnina et al., 2005). Recent findings also elucidated Rho-independent mechanism of Gα_12_-mediated SRE activation via Hsp90 (Figure 1; Montgomery et al., 2014). Interaction between Gα_12_ and Hsp90 might also be critically involved in a selective transport of the G_12_-protein to the lipid rafts (Waheed and Jones, 2002).

Detailed analysis of 5-HT_7_ receptor-mediated signaling revealed that coupling of receptor to the heterotrimeric G_12_-protein selectively activates both RhoA and Cdc42 (Kvachnina et al., 2005), suggesting existence of cross-talk between Cdc42 and RhoA pathways. This might be mediated *via* convergent actions of these GTPases on the downstream effector myosin (Manser et al., 1994; Amano et al., 1996). Alternatively, Cdc42 and RhoA may function in a hierarchical cascade wherein Cdc42 downregulates RhoA activity (Figure 1; Li et al., 2002).

In neuroblastoma cells, agonist-dependent activation of recombinant 5-HT_7_ receptor induces pronounced filopodia formation *via* a Cdc42-mediated pathway paralleled by the RhoA-induced cell rounding (Kvachnina et al., 2005). Stimulation of the 5-HT_7_R/G_12_ signaling pathway in cultured hippocampal neurons promotes formation of dendritic spines and accelerates synaptogenesis, leading to enhanced spontaneous synaptic activity (Kobe et al., 2012). Morphogenic action of 5-HT_7_ receptor was further confirmed in experiments with striatal and cortical neuronal cultures (Speranza et al., 2013). In this study authors observed pronounced neurite outgrowth after specific activation of 5-HT_7_ receptor and demonstrated involvement of ERK and Cdk5 in this process, presuming both proteins to be downstream signaling molecules of Gα_12_ (Speranza et al., 2013).

Noteworthy that 5-HT_7_/G_12_ signaling in hippocampus undergoes strong developmental regulation. In organotypic hippocampal cultures from juvenile mice, 5-HT_7_R/G_12_ signaling potentiates formation of dendritic spines, increases the basal neuronal excitability and modulates synaptic plasticity. In contrast, in older neuronal preparations, stimulation of 5-HT_7_ receptor had no effect on neuronal morphology, synaptogenesis and synaptic plasticity (Kobe et al., 2012). Accordingly, the expression level of both 5-HT_7_ receptor and G_12_-protein in the hippocampus is progressively decreased during postnatal development (Kobe et al., 2012). Thus, 5-HT-induced activation of the 5-HT_7_R/G_12_ signaling pathways and the consequent reorganization of the dendritic morphology appear to be a part of the molecular cascade required for the growth of new synapses and the formation of initial neuronal networks, which then become the subject of activity-dependent structural and functional plasticity (Citri and Malenka, 2008; Ibata et al., 2008).

## Homo- and heterodimerization of 5-HT_7_ receptors

G-protein-coupled receptors were initially assumed to exist and function as monomeric units that interact with corresponding G-proteins in 1:1 stoichiometry. Recent studies revealed the capability of GPCRs to form oligomers (Devi, 2001; Bulenger et al., 2005), and it is now widely accepted that homo- and heterodimerization can represent an additional mechanism regulating GPCR-mediated signaling.

Pharmacological analysis in combination with BRET experiments demonstrated that 5-HT_7_ receptor can form homooligomers in recombinant system (Teitler et al., 2010; Figure 1). Existence of 5-HT_7_ receptor homodimers has also been shown in primary cultures of rat cortical astrocytes (Smith et al., 2011). Homooligomerization of 5-HT_7_ receptor at the single-cell level has been further confirmed using two different FRET assays (Renner et al., 2012).

By combined application of biochemical and biophysical approaches we have recently demonstrated that 5-HT_7_ receptors can form heterodimers with 5-HT_1A_ receptors both *in vitro* and *in vivo* (Renner et al., 2012; Figure 1). From the functional point of view, heterodimerization decreases G_i_-protein coupling of 5-HT_1A_ receptor and attenuates receptor-mediated activation of G-protein-gated potassium (GIRK) channels, without substantial changes in the coupling of 5-HT_7_ receptor to the G_s_-protein. Moreover, heterodimerization significantly facilitated internalization of 5-HT_1A_ receptor, while internalization kinetics of 5-HT_7_ receptor was decelerated upon heterodimerization (Renner et al., 2012).

## Palmitoylation of the 5-HT_7_ receptor

Many signaling molecules involved in GPCR-mediated signaling are modified by post-translational modifications (Escribá et al., 2007), such as phosphorylation, ubiquitination, glycosylation, palmitoylation and others. The experiments with mutations of two predicted N-glycosylation sites in 5-HT_7(a)_ receptor (N5Q and N66Q) revealed, that 5-HT_7(a)_ receptor glycosylation neither influence the binding of 5-CT agonist to the receptor, nor the potency or efficacy with respect to activation of second messenger cascades, although a decrease in receptor density is apparent for the non-glycosylated receptor (Gellynck et al., 2012). To date, no data about the phosphorylation or ubiquitination of 5-HT_7_ receptor are available.

Covalent attachment of long chain saturated fatty acids (i.e., palmitate) to cysteine residue(s) within the protein *via* a labile thioester linkage (S-palmitoylation) represents a widespread post-translational modification of GPCRs since approximately 80% of all known receptors contain the potentially palmitoylable cysteine residue(s) downstream of their seventh transmembrane domain (Escribá et al., 2007). GPCR palmitoylation is involved in the modulation of different receptor functions from coupling to G-proteins and regulation of endocytosis to receptor phosphorylation and desensitization. Also the serotonin receptors represent potential substrates for palmitoylation, and palmitoylation was experimentally demonstrated for 5-HT_1A_, 5-HT_1B_, 5-HT_4_ and 5-HT_7_ receptors (reviewed in Gorinski and Ponimaskin, 2013).

**Figure 2:**
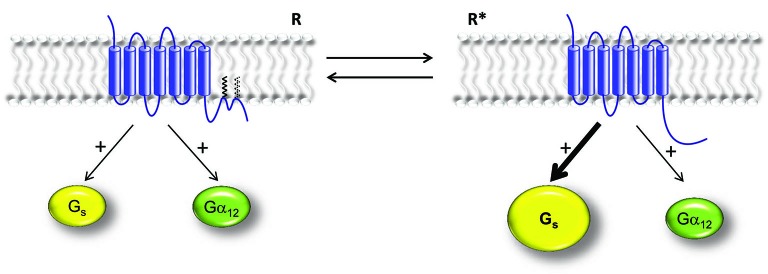
**Hypothetical model for the regulation of 5-HT_7_ receptor activity by dynamic palmitoylation**. This model suggests existence of two different receptor populations: Palmitoylated receptors with two additional intracellular C-terminal loops (left) and non-palmitoylated receptors with no intracellular C-terminal loops (right). These populations exist in dynamic equilibrium regulated by basal or agonist-promoted palmitate turnover. Depalmitoylation results in significant increase of the receptor’s capacity to convert from the inactive (R) to the active (R*) form in the absence of an agonists. Palmitoylated receptor shows activation of both Gα_s_- and Gα_12_-proteins (left). Non-palmitoylated receptor possesses increased agonist-independent, constitutive activity (R*) towards Gα_s_-mediated signaling, while basal receptor-mediated activation of Gα_12_-protein is unaltered (right).

The mouse 5-HT_7_ receptor has been shown to undergo dynamic palmitoylation in an agonist-dependent manner after expression in Sf.9 insect cells. Mutation analysis demonstrated that cysteines located in the C-terminal receptor domain at positions 404, 438 and 441 represent the main potential palmitoylation sites (Figure 2). Although these cysteine residues were responsible for the attachment of more than 90% of the receptor-bound palmitate, palmitoylation of 5-HT_7_ receptor was still not restricted to its C-terminus, pointing to the existence of additional acylation site(s) within the receptor.

Functional analysis of palmitoylation-deficient mutants revealed that agonist-induced activation of G_s_- and G_12_-proteins was unaffected. However, mutation of the Cys404 either alone or in combination with Cys438/Cys441 significantly increased the agonist-independent, G_s_-mediated constitutive 5-HT_7_ receptor activity, while the activation of G_12_-protein was not affected (Figure 2; Kvachnina et al., 2009). Generally, these data suggest that palmitoylation of 5-HT_7_ receptor might be directly involved in the isomerization of the receptor from the inactive to the active form in the absence of agonists. This transformation can be realized by dictating the conformation of receptor’s flexible cytoplasmic loops which might be involved either in the receptor/G_s_-protein recognition or in G_s_-protein binding and/or receptor-mediated G_s_-protein activation (Figure 2). In combination with the previous findings on the functional role of 5-HT_4_ receptor palmitoylation (Ponimaskin et al., 2002, 2005), this observation suggests that palmitoylation can represent a general feature regulating constitutive receptor activity. Moreover, in case of 5-HT_7_ receptor (which is coupled to both, G_s_- and G_12_-proteins) dynamic palmitoylation can represent a molecular mechanism responsible for selective G_s_- or G_12_-mediated signaling.

## Pharmacological properties of 5-HT_7_ receptor

During the last decade, several selective agonists and antagonists for 5-HT_7_ receptors have been developed and applied to investigate its pharmacology. Pharmacological analysis revealed that application of risperidone, 9-OH-risperidone, methiothepin, bromocryptine, lisuride, and metergoline resulted in irreversible inhibition of the recombinant 5-HT_7_ receptor expressed in HEK-293 cells (Smith et al., 2006; Knight et al., 2009). In contrast, action of other potent 5-HT_7_ receptor antagonists, including clozapine, mesulergine, penfluridol, amperozide and cinanserin is reversible and can be washed out (Knight et al., 2009). In other study receptor-inactivating properties of risperidone, 9-OH-risperidone, bromocriptine, methiothepin, metergoline, and lisuride have been demonstrated. Noteworthy that methiothepin and bromocriptine maximally inhibited forskolin-stimulated adenylate cyclase, whereas the other drugs produced partial inhibition, indicating the drugs are inducing slightly different inactive conformations of the 5-HT_7_ receptor (Toohey et al., 2009). Nowadays, the highly specific 5-HT_7_ receptor antagonist SB-269970 (p*K*_i_ = 8.9 nM) is a mostly used receptor antagonist for *in vitro* and *in vivo* studies (Kobe et al., 2012; Renner et al., 2012; Tokarski et al., 2012; Vasefi et al., 2013; Guseva et al., 2014; Monti and Jantos, 2014). For the pharmacological activation of the receptor, a high-affinity receptor agonist 5-CT (IC_50_ = 0.83 nM, EC_50_ 13 nM) is widely used in a numerous *in vitro* and *in vivo* studies (Guscott et al., 2003; Kobe et al., 2012; Vasefi et al., 2013). However, 5-CT is known to activate 5-HT_1A_, 5-HT_1B_, and 5-HT_1D_ receptors. Therefore, analysis of 5-HT_7_ receptor functions by 5-CT requires parallel application of 5-HT_1A/1B/1D_ receptor antagonists. Recently, various novel selective agonists such as AS-19, LP-44, LP-12, LP-211 and E-55888 were developed in addition to 5-CT (reviewed in Di Pilato et al., 2014). Amongst them two novel agonists, LP-211 and LP-378, have been investigated in regard to exploratory motivation, anxiety-related profiles, and spontaneous circadian rhythm (Adriani et al., 2012). The authors have shown that three- to four-fold dosage of LP-378 was necessary to induce the same effect as LP-211. The latest studies, both *in vitro* and *in vivo*, indicated LP-211 (*K*_i_ = 379 nM) as a more specific 5-HT_7_ receptor agonist with great potential for future investigations (Speranza et al., 2013; Monti and Jantos, 2014).

## Conflict of interest statement

The authors declare that the research was conducted in the absence of any commercial or financial relationships that could be construed as a potential conflict of interest.
